# Translating Molecules into Imaging—The Development of New PET Tracers for Patients with Melanoma

**DOI:** 10.3390/diagnostics12051116

**Published:** 2022-04-29

**Authors:** Laetitia Vercellino, Dorine de Jong, Laurent Dercle, Benoit Hosten, Brian Braumuller, Jeeban Paul Das, Aileen Deng, Antoine Moya-Plana, Camry A’Keen, Randy Yeh, Pascal Merlet, Barouyr Baroudjian, Mary M. Salvatore, Kathleen M. Capaccione

**Affiliations:** 1 Nuclear Medicine Department, University Hospital Saint-Louis, Assistance Publique-Hôpitaux de Paris, Université Paris Cité and INSERM, UMR_S942 MASCOT, 75006 Paris, France; laetitia.vercellino@aphp.fr (L.V.); pascal.merlet@aphp.fr (P.M.); barouyr.baroudjian@aphp.fr (B.B.); 2 Center for Cell Engineering, Memorial Sloan Kettering Cancer Center, New York, NY 10065, USA; ddejong990@gmail.com; 3 Department of Radiology, Columbia University Irving Medical Center, New York, NY 10032, USA; ld2752@cumc.columbia.edu (L.D.); braumuller.b@northeastern.edu (B.B.); cva7191@nyu.edu (C.A.); ms5680@cumc.columbia.edu (M.M.S.); 4 Faculté de Pharmacie de Paris, Université de Paris, Inserm UMR-S1144-Optimisation Thérapeutique en Neuropsychopharmacologie, 4 Avenue de l’Observatoire, 75006 Paris, France; benoit.hosten@aphp.fr; 5 Unité Claude Kellershohn, Institut de Recherche Saint-Louis, Université de Paris, 75013 Paris, France; 6 Service Pharmacie, Assistance Publique-Hôpitaux de Paris (AP-HP), 75005 Paris, France; 7 Department of Radiology, Memorial Sloan Kettering Cancer Center, New York, NY 10065, USA; dasj@mskcc.org (J.P.D.); yehr@mskcc.org (R.Y.); 8 Novant Health Cancer Institute, Charlotte, NC 28144, USA; adeng87@gmail.com; 9 Head and Neck Oncology Department, Gustave Roussy Cancer Campus, 94805 Villejuif, France; antoinemoya@gmail.com; 10 Inserm U981, Paris Saclay University, 91190 Villejuif, France

**Keywords:** PET imaging, melanoma, molecular targeting

## Abstract

Melanoma is a deadly disease that often exhibits relentless progression and can have both early and late metastases. Recent advances in immunotherapy and targeted therapy have dramatically increased patient survival for patients with melanoma. Similar advances in molecular targeted PET imaging can identify molecular pathways that promote disease progression and therefore offer physiological information. Thus, they can be used to assess prognosis, tumor heterogeneity, and identify instances of treatment failure. Numerous agents tested preclinically and clinically demonstrate promising results with high tumor-to-background ratios in both primary and metastatic melanoma tumors. Here, we detail the development and testing of multiple molecular targeted PET-imaging agents, including agents for general oncological imaging and those specifically for PET imaging of melanoma. Of the numerous radiopharmaceuticals evaluated for this purpose, several have made it to clinical trials and showed promising results. Ultimately, these agents may become the standard of care for melanoma imaging if they are able to demonstrate micrometastatic disease and thus provide more accurate information for staging. Furthermore, these agents provide a more accurate way to monitor response to therapy. Patients will be able to receive treatment based on tumor uptake characteristics and may be able to be treated earlier for lesions that with traditional imaging would be subclinical, overall leading to improved outcomes for patients.

## 1. Introduction

Melanoma of the skin is the deadliest form of skin cancer and can also be found in other organs including the eyes and mucosa. The lifetime risk of being diagnosed with melanoma is now 1 in 54 individuals [1]. Prior to the advent of targeted and immunotherapies, stage IV disease had a dismal prognosis, with a median survival time of 7.5 months and a five-year survival rate of approximately 6% [2,3]. 

## 2. Recent Paradigm Shift in the Systemic Therapeutic Armamentarium

Dacarbazine was previously the standard of care therapy in metastatic melanoma [4], with response rates of approximately 20% but with a dismal five-year overall survival rate of less than 10% [5,6,7,8]. Imaging plays a key role in staging melanoma with CT of the chest, abdomen, and pelvis and/or whole body.^18^F-FDG PET-CT most is commonly performed to evaluate patients for metastatic disease. ^18^F-FDG PET-CT is also used to evaluate progression of disease and response to therapy [9]. Research has shown PET-CT to have superior sensitivity for the detection of melanoma metastases, with sensitivity of 94.2% compared to 55.3% for CT alone [10]. More recently targeted inhibitors, especially those against BRAF and MEK inhibitors, have emerged as important therapeutic agents in melanoma therapy. A significant portion of cutaneous melanomas have a BRAF mutation, which has led to the development of drugs targeting proteins produced by this mutant gene [11,12]. Two BRAF inhibitors, vemurafenib and dabrafenib, are approved in the United States and Europe for treatment of patients with unresectable or metastatic melanoma with BRAF V600E mutations and function as small molecule inhibitors of BRAF kinase [13]. The BRIM-3 trial examined the efficacy of vemurafenib for the treatment of metastatic melanoma, and found an overall 84% six-month survival rate compared to a 64% survival rate in those treated with dacarbazine. Vemurafenib was also associated with increased overall and progression-free survival [14]. Similarly, MEK inhibitors have offered hope to patients with melanoma. Mitogen-activated protein kinase (MEK) 1/2 are downstream targets of BRAF and drive oncogenesis in melanoma. An early study by Flaherty et al. demonstrated that administration of the MEK inhibitor trametinib improved overall survival and progression-free survival compared to those treated with dacarbazine [15]. Subsequently, several studies evaluated combined BRAF and MEK inhibition and found improved outcomes with the combined regimen compared to either alone [16]. 

The advent of checkpoint inhibitor immunotherapy represented a major advance in the treatment of melanoma. In 2010, a seminal study in the New England Journal of Medicine using the CTLA-4 inhibitor ipilimumab demonstrated an increased overall survival rate of 10.1 months compared to 6.4 months when patients were treated with an experimental peptide vaccine alone. Importantly, researchers demonstrated that approximately 20% of patients achieved a durable response [17]. A meta-analysis by the same group assessing response rates to ipilimumab in patients with unresectable or metastatic melanoma in 12 studies, including both prospective and retrospective research, confirmed a durable response in approximately 20% of patients [18]. PD-1 inhibition provided another opportunity to modulate the immune activation pathways, increasing the number of patients achieving a durable response. A head-to-head study of PD-1 inhibitor pembrolizumab and ipilimumab demonstrated that pembrolizumab resulted in a 46–47% six-month progression-free response compared to ipilimumab, which demonstrated a 27% progression-free response [19]. Ultimately, research demonstrated that combined CTLA-4 and PD-1 blockade was more efficacious than ipilimumab alone, leading to FDA approval of combined treatment for melanoma [20,21].

Immunotherapies are an important systemic therapy option for patients with metastatic or unresectable melanoma and have emerged as the preferred first-line treatment in most melanoma populations in various guidelines [22,23]. This includes its use as an initial treatment for treatment-naïve patients with metastatic melanoma with or without BRAF V600 mutation where immunotherapy agents have been associated with durable long-term survival in patients with responding disease.

## 3. Current Role of Medical Imaging

In patients with a diagnosis of melanoma, medical imaging is essential for the tumor stage assessment, the evaluation of prognosis, and defining the therapeutic algorithm. Currently, for cutaneous melanoma (CM), T stage is only defined on primary tumor pathological analysis according to Breslow thickness and ulceration. Nodal involvement is one of the main prognostic factors in melanoma as N+ patients are considered as stage III by the Union for International Cancer Control (UICC), with a high risk of distant metastases, requiring adjuvant immunotherapy [24]. For cN0 patients both clinically and radiologically (CT scan and/or ultrasound), sentinel node (SN) biopsy coupled with SPECT-CT is the standard of care to evaluate the potential nodal involvement [25]. SN status remains the strongest prognostic factor for patients with CM [26].

In CM treatment, surgery is not only required for primary tumor resection with clear margins, but also for tumor staging for the evaluation of nodal involvement in cN0 patients. Improving the accuracy of SN detection with better pre-operative visualization on SPECT-CT may be useful to limit the rate of false negative cases. Moreover, in some specific regions such as the head and neck, SN detection may be more difficult due to “shine-through” radioactivity of the primary tumor, which could mask the positive lymph nodes in close proximity [27]. Therefore, new detection methods of SNs are needed, including the use of new tracers that rapidly clear the injection site but would be selectively retained within the SN [28]. 

Considering the surgical management of patients with mucosal melanoma (MM), two main issues remain: the poor prognosis linked to the disease’s high metastatic potential and the morbidity related to the primary tumor resection [29]. The decision of tumor resection feasibility and morbidity often rely heavily on CT or MR imaging. However, current imaging techniques are limited in their ability to visualize melanoma given that it is often multifocal with superficial and mucosal extensions that are not well visualized on these modalities or PET-CT [29,30,31]. Tumor resection feasibility and morbidity are determined according to CT-scan and MRI analysis. Combining imaging techniques, such as PET-MRI with specific melanoma tracers may increase sensitivity in detecting the extent of the disease that can more precisely guide surgical planning and avoid unnecessary surgeries in patients with unresectable disease. Moreover, an earlier detection of patients with metastatic disease and/or locally advanced tumors could be very useful for avoiding unnecessary surgery (e.g., maxillectomy).

Recently, specific tumor characteristics on ^18^F-FDG PET-CT were described as prognostic biomarkers of oncological outcomes and response to immune checkpoint inhibitors [32]. Thus, these imaging biomarkers could be of primary importance in choosing between local treatment and immunotherapy. In the case of neo-adjuvant therapy (e.g., immunotherapy or targeted therapy), the imaging response assessment with the distinction between tumor necrosis, “flare” phenomena and residual disease constitute another challenge to address in order to adapt the therapeutic strategy and determine the ideal timing for surgery.

While PET-CT and CT imaging are the standard of care for staging and monitoring the progression of melanoma, other imaging modalities may be used for problem solving or to provide supplemental information. One such modality is high frequency ultrasound (HFUS), or ultra-high frequency ultrasound (ultra-HFU). Because of the high resolution of superficial structures, studies have shown that HFUS can determine the Breslow index in a significant number of patients, providing important clinical information previously only obtained via biopsy [33,34]. Another recently developed approach is the use of multispectral imaging for the diagnosis of melanoma. A prospective study using a multispectral imaging system on 1391 skin lesions, including 184 melanoma lesions, demonstrated that with the use of a neural network trained to automate diagnosis, the network was able to discriminate between non-melanomatous lesions and melanoma with a sensitivity of 80.4% and a specificity of 75.6% using histology as the gold standard diagnosis [35]. While neither of these techniques have reached widespread use, they offer promise to supplement PET-CT and CT imaging in cases where these traditional modalities are non-diagnostic. 

Despite advances, progressive or recurrent disease remains a major concern for patients with melanoma. Sentinel lymph node biopsy is widely used to stage the disease and it assesses both micrometastatic and macrometastatic disease [36]. While most research has focused on metastatic disease in sentinel lymph nodes [37,38,39], disseminated micrometastatic disease including lesions less than 2 mm in greatest dimension, which is below the resolution of traditional imaging, may have a significant clinical impact and is currently not well identified. Similarly, metastatic dormancy in melanoma has been observed in both cutaneous and uveal melanoma, representing a clinical risk but also an area of opportunity to improve outcomes [40]. The ability to identify micrometastatic or dormant metastatic disease that is below the resolution of current CT or PET-CT would constitute a major advance in the field and allow for more accurate staging and monitoring of the progression of the disease. Targeted molecular imaging represents the best strategy for identifying disease that is not visualized on traditional imaging modalities. Thus, there has been keen interest in developing targeted molecular imaging agents to better visualize micrometastatic disease. Numerous cancer-specific or melanoma-specific tracers are in preclinical development and may change the landscape of melanoma diagnosis and monitoring. Here, we detail some of the most promising new agents and describe their progress toward approval for clinical use.

## 4. Clinical Challenges and Unmet Need

Imaging is critical to assess the extent of disease, detect recurrence, or monitor disease progression and therapeutic response. Different imaging techniques pose their own clinical challenges with limitations in sensitivity and specificity and risks of both false-positive and false-negative results. While false-positive findings may result in unnecessary and invasive procedures, false-negative findings may result in delayed diagnosis and treatment. In one study of 154 patients with stage III melanoma who underwent surveillance using CT or PET-CT, the false positive rate was 53%, with 88% of lesions found to be benign [41]. Non-malignant conditions such as benign skin conditions, post-vaccine nodal uptake, and treatment-related findings such as that seen with immunotherapy can limit accurate imaging interpretation [42]. Furthermore, PET-CT imaging uses ionizing radiation both via radiotracer administration and the CT portion of image acquisition, posing a small, but nevertheless non-zero risk to the patient. Compared to other cancers, melanomas often occur in patients at a younger age and therefore the cumulative risk of radiation exposure over time may become significant with lifetime post-treatment surveillance. Given these limitations, there remains an ongoing need to develop imaging modalities that are both highly sensitive and specific in detecting melanoma.

## 5. PET Imaging Agents for Cancer

**Figure 1:**
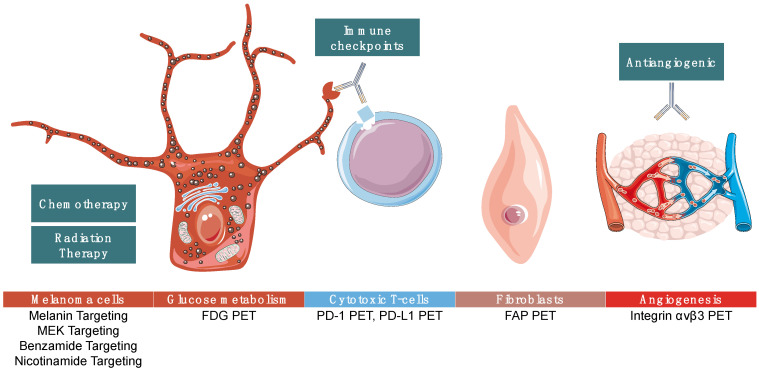
Summarizes targets of positron emission tomography (PET) imaging agents that are in various stages of development as discussed below. Some agents are melanoma-specific while others can be used in many cancer types.

Many new PET imaging agents developed for cancer imaging can be used in patients with melanoma and this strategy may address many limitations of current imaging, providing prognostic information and mapping of tumor heterogeneity, and may help detect lesions below the resolution of CT imaging. Here, we present several of the most promising new imaging agents that may transform the diagnosis, staging, and monitoring of metastatic melanoma. We focus on novel imaging agents; however, often there is a companion therapeutic treatment using the same targeting molecule and a therapy radionuclide, which we briefly touch upon in the text. Figure 1 summarizes PET imaging targets to illustrate the melanoma discussed in this paper.

## 6. ^18^F-FDG Targeting

^18^F-FDG PET is a “non-melanoma” specific agent that has demonstrated its usefulness for several indications such as staging, prognostication, and treatment response assessment, and it may have theranostic applications [43]. Its main advantages are its cost, availability, FDA approval, and the fact that most non-resectable melanoma tends to be ^18^F-FDG avid, including cutaneous, mucous, and uveal melanoma [32]. 

Its main limitations are two-fold. First, it has limited utility for the detection of subcentimeter lesions and hence limited performance for the detection of micrometastases [43]. Second, ^18^F-FDG avidity is not a specific biomarker since glucose metabolism is increased in a wide range of inflammatory processes. For instance, one of the challenges is that new ^18^F-FDG avid lesions may represent potentially life-threatening treatment-induced, immune-related adverse events which should be promptly diagnosed to initiate dedicated treatment [44,45]. 

^18^F-FDG PET-CT has been demonstrated to be a powerful prognostication tool. Several studies have demonstrated that increased tumor volume and increased glucose metabolism in hematopoietic tissues (i.e., spleen and bone marrow) are typically associated with poor prognosis, lower response rate, and an immunosuppressive environment in cutaneous, uveal, and mucosal melanoma. In non-resectable mucosal melanoma, high baseline maximal glycolytic activity (SUVmax) was correlated with poorer outcomes in immunotherapy [32]. In non-resectable cutaneous melanoma, high baseline metabolic tumor burden and high bone marrow metabolism were correlated with poorer outcomes in immunotherapy [46]. In a literature review, the prognostic and predictive value of spleen glucose metabolism has been demonstrated. High spleen glucose metabolism at baseline was associated with a poor outcome while treatment-induced change in spleen glucose metabolism was a more equivocal imaging biomarker [47]. Strikingly, biomarkers derived from the analysis of hematopoietic tissues seemed not only a strong prognosticator of outcome but also are reproducible metrics [48]. 

For treatment response assessment, the pivotal role that ^18^F-FDG PET-CT is playing in the field of immunotherapy is demonstrated by the multiple response criteria that have been developed and then finely tuned by the medical community, such as PECRIT, PERCIMT, imPERCIST, and iPERCIST [49,50]. There is growing evidence that FDG PET-CT outperforms conventional imaging with CT-scan for early prediction of response and to provide an early readout on survival in melanoma [51,52,53,54].

Since ^18^F-FDG PET-CT is the current standard of care for molecular imaging of patients with a diagnosis of melanoma, the emerging imaging techniques will need to demonstrate how they can outperform and provide additional value to this current benchmark.

## 7. PD-1/PD-L1 Targeting

PD-1 and PD-L1 inhibitors have been approved by the FDA and EMA for management of advanced and metastatic melanoma as well as other types of cancer and have paved the way for radiolabeled agents for imaging using these antibodies. These agents now constitute first line therapy in most patients, alone or in combination with ipilimumab, an anti-CTLA-4 molecule, even in those with BRAF mutation [23]. About 40% of patients will have a durable response, but no existing biomarkers can accurately predict which patients will respond to therapy at this time. One option to attempt to predict response is to perform anti-PD-1 and/or anti-PDL-1 immunohistochemistry; however, this approach has limitations related to sample heterogeneity from sampling bias and differences in staining techniques. Imaging PD-L1 could overcome some of these limitations and potentially show micrometastatic lesions that are PD-1/PD-L1 positive [55]. Furthermore, longitudinal imaging of these targets might be useful for providing an early and reliable assessment of therapeutic response. Several radiotracers targeting PD-1 and PD-L1 have been developed and are at varying levels of preclinical and clinical development, with some advancing to proof-of-concept studies in humans.

PD-1 and PD-L1 inhibitors are monoclonal antibodies with multiple sites that may be radiolabeled for imaging. The prolonged clearance time of these molecules from the circulation, which takes several days, requires the use of radionuclides with long half-lives. Thus, radionuclides that are more commonly used to image full PD-1/PD-L1 molecules include ^89^Zr (half-life: 78 h) or ^64^Cu (half-life: 12.7 h). Researchers have found that ^89^Zr labelled recombinant human antibody targeting an extracellular domain on human and mouse PD-L1 could evaluate the PD-L1 levels in xenografts derived from non-small cell lung cancer (NSCLC) that subsequently responded to PD-L1 blockade [56]. The optimal time point for imaging was 48 hours post-injection. The group also demonstrated that the radiotracer could detect low levels of PD-L1 in xenografts of other tumor types and therefore could be used to quantify changes of PD-L1 expression resulting from treatment [56].

A radiolabeled monoclonal antibody which is similar to atezolizumab, ^89^Zr-DFO-6E11, has been used for imaging of PD-L1 positive tumors, identifying levels of PD-L1 expression and variation of PD-L1 expression with treatment. Tumors with the highest uptake responded better to therapy [57]. Another study evaluated an ^89^Zr labeled anti-mouse PD-L1 antibody fragment for *in vivo* assessment of PD-L1 levels in a melanoma mouse model. This agent demonstrated better imaging qualities than the full radiolabeled antibody, with earlier and higher uptake in tumors expressing PD-L1 at 2, 24, and 48 hours [58]. A separate study of ^64^Cu-atezolizumab showed PD-L1 expression across 5 different tumor cell lines, with imaging performed at 24 and 48 hours [59]. While these results have been encouraging, the delay between injection and imaging necessary for imaging these agents may be suboptimal for clinical use, and their dosimetry has been shown to be less favorable than for other agents utilizing radionuclides with shorter half-lives such as ^18^F and ^68^Ga. The use of small molecules targeting PD-1 or PD-L1, such as antibody fragments, small proteins, or peptides, confers the possibility of using a radionuclide with a shorter half-life, thereby allowing for same day imaging [60].

The imaging properties of ^68^Ga make it an attractive option for PD-1/PD-L1 imaging. Nb109 is a non-blocking single-domain anti-PD-L1 antibody. A study evaluated ^68^Ga-NOTA-Nb109 uptake using mice bearing tumors generated from the human melanoma cell line A375 transfected with the human PD-L1 gene. Their data demonstrated that the optimal imaging time was 1 hour after injection and that uptake in tumors correlated with PD-L1 expression as assessed by immunohistochemistry. The same radiotracer, ^68^Ga-NOTA-Nb109, was evaluated in another preclinical study of non-melanoma tumors and demonstrated changes in PD-L1 expression in response to cisplatin therapy, which could provide clinically useful information in patients undergoing therapy [61].

Although many tracers have been evaluated in preclinical models, few have led to first-in-human studies. Researchers assessed clinical ^89^Zr-atezolizumab PET-CT imaging in 22 patients with different tumor types before initiation of atezolizumab therapy [62]. The injection was safe and generally well-tolerated. The optimal timepoints for imaging were 4 and 7 days after tracer injection. Analysis of biodistribution of ^89^Zr-atezolizumab demonstrated uptake in non-malignant lymph nodes, lymphoid tissues, spleen, and at sites of inflammation. Radiotracer uptake was heterogeneous and varied with the site of the lesion and the type of cancer. Heterogeneity was also observed across the different lesions within the same patient. Patients with higher uptake values had a better objective treatment response. They also had better progression-free and overall survival. ^89^Zr-atezolizumab better predicted patients’ outcomes than immunohistochemistry performed for comparative analysis [62].

PD-L1 PET imaging using ^18^F-BMS-986192 [60] and PD-1 PET imaging with ^89^Zr-nivolumab [63] has also been further evaluated in clinical studies. The optimal imaging time points were 70–90 min after injection of ^18^F-BMS-986192 and 7 days after injection of ^89^Zr-nivolumab. Radiotracer uptake was heterogeneous with both radiopharmaceuticals, varying between patients and among lesions within the same patient. Uptake intensity correlated with PD-1 or PD-L1 expression as determined by immunohistochemistry. For both agents, lesions with the highest uptake as assessed by SUVpeak responded better to checkpoint inhibitor therapy. 

^124^I-JS001 is a radiolabeled toripalimab, a humanized monoclonal antibody against the PD-1 receptor that has been used for PET-CT and PET-MR imaging in patients with melanoma and urologic cancers [64]. Biodistribution and uptake was studied at 24 and 48 hours post-injection. Primary tumors and metastatic lesions showed different levels of uptake, with intra- and inter-individual heterogeneity. Interestingly, two patients previously treated by toripalimab showed no lesion uptake, suggesting that this agent may have the potential to monitor if toripalimab has reached a target lesion. While this study confirmed the potential of ^124^I-toripalimab as a PET imaging agent, it lacked correlation with PD-1 immunohistochemical staining and did not evaluate response to treatment, and thus the agent warrants further investigation before it could be considered for clinical use. 

A prospective phase I study evaluated ^68^Ga-NOTA-WL12, a radiolabeled small peptide with a high affinity with PD-L1 in 9 patients with immunohistochemistry-positive PD-L1 non-small cell lung cancers, along with ^18^F-FDG PET [65]. These patients were subsequently treated with a combination of pembrolizumab and chemotherapy [65]. Tumors were identified as soon as one hour post-injection. Furthermore, the injection was safe and well tolerated with significant hepatobiliary clearance and urinary excretion. Patients with high PD-L1 expression had higher radiotracer uptake than patients with low PD-L1 expression, and there was a strong correlation between most ^68^Ga-NOTA-WL12 PET uptake measures and PD-L1 expression, whereas only SUVmean uptake values of ^18^F-FDG PET correlated with PD-L1 expression.

While much work remains to be done, these proof-of-concept studies offer great prospects for the future of PD-1- and PD-L1-targeted PET imaging agents in melanoma for the prediction of response to immune checkpoint inhibitors. Further large-scale trials are needed before approval for clinical use. Several clinical trials are ongoing worldwide for these agents, recruiting patients with multiple types of cancer, including melanoma. These studies may provide evidence for the widespread use of PD-1- and PD-L1-targeted PET imaging agents [66].

## 8. FAP Targeting

The development of fibroblast activation protein (FAP) imaging has represented a major advance in oncological PET imaging given that it can be used to image many tumor types. It has proven to be an excellent imaging agent particularly in tumors that are poorly imaged by FDG-PET, owing to the ubiquitous nature of fibroblasts in cancer. Given that cancer associated fibroblasts (CAFs) are disease specific, there is high tumor-to-background uptake. The main limitation of FAP-targeting ligands is that they also image other disease states that result in fibroblast activation, such as pulmonary fibrosis. Despite this, FAP-targeted PET imaging is highly promising for the detection of primary and metastatic disease. 

Fibroblast activation protein (FAP) is a serine protease expressed in activated fibroblasts that infiltrate tumors and provide support for cancer cells [67,68]. Given that fibroblasts are ubiquitous within tumors, scientists recognized that targeting FAP would have great potential for imaging tumors and for the delivery of molecular targeted radiotherapy [69,70]. In 2017, researchers published their work optimizing FAP targeting molecules. Using a quinolone-based backbone, they made a series of 15 compounds with slight variations in chemical moieties. They characterized each of these in terms of binding, internalization, and preclinical pharmacokinetics in order to identify a lead compound which was ultimately FAPI-04. The group performed first-in-human studies of this molecule conjugated to ^68^Ga (^68^Ga-FAPI-04), imaging two patients with metastatic breast cancer, and demonstrating high uptake in metastasis. They also conjugated FAPI-04 to ^90^Y (^90^Y-FAPI-04) and administered this compound, which resulted in palliation of pain associated with metastasis [61]. 

Given the success of FAPI-04, the group went on to demonstrate ^68^Ga-FAPI-04 PET imaging in 28 types of cancer, including some cancers—such as small intestine cancer—that are difficult to image on ^18^FDG-PET [71]. They further evaluated ^68^Ga-FAPI-04 PET as an imaging agent for rare tumors, defined as tumors having an incidence of less than 1 in 2000, and found that it was an effective PET imaging agent for the primary tumor in approximately 20% of these tumors and more than 50% of metastatic lesions [72]. A separate group validated the use of ^18^F-FAPI-04 PET as a comparable alternative to ^68^Ga-FAPI-04 PET [70]. Given the avidity of FAP for tumors as demonstrated by the remarkable success of FAP imaging agents, newer compounds including FAPI-42 are being developed to increase tumor retention time [73]. More recently, clinical FAP-targeted PET imaging has been used to image lymphoma in addition to solid tumors [74]. Other FAP-targeting imaging agents such as (4-quinolinoyl)-glycyl-2-cyanopyrrolidine-based small molecules [75,76] and FAP-2286 [77] are also in development; however, they have not yet been as widely used as FAPI-04.

Early clinical research studies have now given way to larger studies evaluating FAP-targeted imaging agents. An ongoing clinical trial (NCT04441606) is currently enrolling patients with the goal of evaluating ^68^Ga-FAPI-04 PET compared to ^18^FDG-PET to evaluate disease burden, with an emphasis on malignancies with variable ^18^FDG uptake [78]. Another clinical trial (NCT04499365) plans to enroll 500 patients for ^68^Ga-FAPI-04 PET to provide robust clinical data regarding the use of FAP-targeted imaging to diagnose and monitor cancer [79]. Several FAP-targeted radiotherapies are in clinical trials and others are in preclinical stages of development. Given the ubiquitous nature of fibroblasts in cancer and the avidity of FAP-targeted imaging agents, FAP-targeted PET imaging may significantly change the landscape of oncological PET imaging.

## 9. Melanin Targeting

Melanin imaging is specific to melanoma and has demonstrated its usefulness for several applications such as early diagnosis, staging, progression monitoring, and management of both local and disseminated melanoma. The main advantages are its broad applicability to all pigmented melanoma patients, its high specificity, and the potential to use melanin-targeted radionuclide therapy. Its limitations are potential off-target toxicities. Recent studies have demonstrated that targeting melanin may be more complicated than originally thought, given the plasticity of melanoma cells, the wide range of cell states, and differences in gene expression in patient-derived cell samples compared to cell lines, which have traditionally been used in many research studies. A recent study evaluating patient-derived samples demonstrated that tyrosine levels, a precursor in melanin synthesis, promoted phenotypic drift towards a mesenchymal phenotype and was associated with resistance to MAPK inhibitors [80]. Overall, this and other studies have highlighted the complexity of melanin targeting and its effects on melanocytes, which implies that while it may serve as a target for melanoma, its role is complex and may complicate the use of these inhibitors clinically [81].

The pigment melanin is endogenously synthesized by melanocytes in multiple steps and is present in many tissues of the human body, including the epidermis, hair follicles, eyes, inner ear, bones, heart, substantia nigra, locus coeruleus, and leptomeninges [82,83,84]. Melanin molecules are biopolymers with non-hydrolysable bonds and have both homeostatic and protective functions, including thermoregulation, photoregulation, and protection against UV damage. Most melanomas also produce melanin. High melanin content in melanoma cells has been correlated to disease progression and lower survival as it is involved in immunosuppression and resistance to chemotherapy and radiotherapy [85,86]. Given that 90% of melanomas express melanin, it is an attractive molecular target for PET imaging of melanoma [87], and because it is highly overexpressed in pigmented melanoma, melanin-binding probes can be excellent imaging agents for early detection of the disease [88]. As a companion therapeutic, melanin-targeted radionuclide therapy has demonstrated promising therapeutic responses *in vivo* [89,90]. Given the promise of melanin-targeting imaging and therapy radionuclides, researchers have worked to develop and optimize these agents to have improved tumor uptake and retention, excellent tumor-to-background ratio, and low toxicities with rapid renal clearance.

Melanin-targeting tracers have the potential to address current shortcomings in detecting melanoma microlesions and are easy and cost effective to produce on site; in particular, three tracers have demonstrated *in vivo* imaging performance superior to ^18^F-FDG: ^18^F-5-FPN, which is also called ^18^F-P3BZA [91,92,93,94], 18F-DMPY2 [95], and ^18^F-ICF01006, which is also called ^18^F-MEL050 [88,96,97,98]. ^18^F-5-FPN (^18^F-5-fluoro-N-[2-(diethylamino)ethyl] picolinamide) synthesis has been optimized and has shown significantly higher uptake in B16F10 cells than ^18^F-FDG (13.29 ± 3.80% ID/g compared to 7.24 ± 1.95%) [91]. Lung metastases less than 2 mm were better visualized with ^18^F-5-FPN than ^18^F-FDG, allowing earlier detection of both regional and distant metastases in mice [95,98]. The radiotracer was also tested clinically in a pilot study with healthy volunteers and in patients with pigmented melanoma, who received ^18^F-5-FPN with an average dose of 5.72 ± 0.42 mCi. Despite the small cohort, results were concordant with preclinical studies demonstrating superior imaging capacity for subcentimeter lesions and no serious adverse effects. In addition, the whole-body effective dose received was less than standard ^18^F-FDG imaging [92].

DMPY2 (N-(2-(dimethylamino)ethyl)-5-^18^F-fluoropicolinamide) resembles ^18^F-5-FPN but contains a reduced alkyl chain bearing the amine residue. It also presents high cell uptake and retention, with more than 103-fold higher uptake in B16F10 melanoma cells compared to control cells [95]. In primary tumors, the tracer can be visualized as early as 30 min post-injection with tumor retention up to 4 hours and extremely low background activity 2 hours after injection. DMPY2 uptake in lung metastasis was 115-fold higher than in normal lung tissue. Given that many tumors categorized as amelanotic are actually hypomelanotic, this excellent imaging quality with prolonged tumor retention time and high signal-to-background ratio suggests that it may be clinically relevant for identifying micrometastatic lesions in these cases. 

^18^F-MEL050 is a benzamine-based agent used for imaging melanin that is detailed below. Together, these melanin-targeting agents have demonstrated high specificity and avidity for melanin, with superior lesion identification *in vivo* compared to ^18^F-FDG. Targeting melanin allows earlier detection of primary lesions and micrometastases that can be occult on ^18^F-FDG PET. Unlike other benzamides such as ^18^F-FBZA [99], these compounds did not show dose-limiting toxicities. Given that melanin expression is a predictor of therapy response, these probes could be used as predictive biomarkers to determine which patients will respond to therapy. Additional studies are needed to determine the best compound and define the threshold of detection. 

As above, targeting melanin for therapy is also a rational strategy that has demonstrated early promise. A group utilized antibodies to treat melanin generated initially for studying *Cryptococcus neoformans* melanogenesis, and they were repurposed for radioimmunotherapy [100]. They radiolabeled this antibody with ^188^Re, which is both a beta and gamma emitter and therefore could be both an imaging and a therapy agent, and demonstrated in a preclinical murine model of melanoma that tumor growth was reduced [101]. This compound was subsequently translated to early clinical trials and showed antitumor efficacy with no dose-limiting toxicities [102]. Given these initial results, more in-depth study is needed to evaluate if this melanin-targeted radiotherapy is appropriate for widespread clinical use.

A concern regarding melanin-targeting molecules is that they may have increased toxicities because of their greater dissemination and retention throughout the body within different sites of metastases. However, studies of ^131^I-ICF01012 and ^131^I-ICF15002 have demonstrated promising anti-tumor results *in vivo* with limited toxicities [86,103].

## 10. Benzamide Targeting

Benzamine imaging is melanoma-specific and has demonstrated its usefulness for several indications such as accurate detection of melanoma metastasis, staging, and evaluation of new treatment strategies. The mechanism of action is based on binding to melanin with a strong affinity in melanoma cells and melanocytes [104].

The main advantages are early, sensitive, and specific detection of pigmented malignant melanoma. Indeed, ^18^F-FDG PET imaging, routinely used for initial staging of III/IV malignant melanoma, is not sensitive for the detection of local and lymph node metastasis [97]. Sensitivity and specificity for detecting melanoma metastases depend on the level of melanin expression, which may change depending on the stage of disease development [86].

Benzamide-based PET imaging agents are another rational strategy for PET imaging of melanoma. Iodobenzamides have been shown to be good candidates for this application given that they have a strong affinity for melanoma [105]. Early studies investigated the influence of structural variations on the biodistribution of six isomers of N-(N-dialkylaminoethylene)-^123^I-iodobenzamide (ABA) in melanoma-bearing murine models. Among these isomers, o-^123^I-ABA 2-2 exhibited the most favorable pharmacokinetic properties as a melanoma imaging agent [106]. Subsequent preclinical studies have demonstrated radiotracer uptake and imaging in a murine model of melanoma after administration of N-(2-diethylaminoethyl)-4-iodobenzamide (^125^I-BZA) in tumor-bearing mice [107]. Its ^123^I analogue (^123^I-BZA) has also been translated for human use and was evaluated in a phase 2 clinical trial as an imaging agent for the detection of primary and metastatic melanoma [108].

More recently, another phase 2 clinical trial studied an ^123^I-BZA isomer, ^123^I-BZA(2). Compared to ^123^I-BZA, ^123^I-BZA(2) showed a similar affinity associated with better clearance allowing for scintigraphy to be more easily performed (2–4 hours after injection) in patients with ocular melanoma and melanin-expressing metastasis [98]. Another clinical trial evaluating ^123^I-BZA(2) showed a sensitivity of 100% and a specificity of 95% for the detection of melanoma lesions in histologically proven cutaneous melanoma patients with or without secondary metastases [109]. A prospective and multicentric phase 3 clinical study compared the sensitivity and specificity of ^18^F-FDG and ^123^I-BZA(2) for the diagnosis of melanoma metastasis in 87 patients with a history of cutaneous or ocular melanoma. While the specificity of detection was not significantly different between ^18^F-FDG and ^123^I-BZA(2) (78% vs. 94%, respectively), the study was ultimately terminated early given the higher sensitivity of ^18^F-FDG compared to ^123^I BZA(2) for diagnosis of melanoma metastases (87% vs. 39% respectively) [110].

More recently, researchers have reported the synthesis and preclinical evaluation of a 4-^11^C-methoxy N-(2-diethylaminoethyl) benzamide (4-^11^C-MBZA) for melanoma PET imaging [104]. As a melanoma PET imaging agent, 4-^11^C-MBZA displayed favorable properties compared to its ^18^F analogues with low uptake in normal tissue and rapid and persistent accumulation in tumors, resulting in a high tumor-to-background ratio [111].

A new method of ^18^F radiolabeling based on coordination chemistry with Al-^18^F complexes is being increasingly used. This method presents several advantages: faster total synthesis time, higher radiolabeling yield, and a simpler radiolabeling process [112,113]. A novel NOTA-benzamide derivative, Al-^18^F-NOTA-BZA, has been developed with this radiolabeling strategy [114]. MicroPET-CT imaging in two melanoma-bearing mouse models displayed positive pharmacokinetic proprieties, including rapid clearance of Al-^18^F-NOTA-BZA from the normal tissues and high uptake within the tumor compared to normal tissue [114].

Another line of research into benzamine-based PET imaging agents for melanoma imaging investigated the radiopharmaceutical (^18^F-MEL050) [33]. It was used as a PET tracer for pigmented melanoma after radiolabeling with ^18^F in experimental models [96,98]. *In vivo* PET-CT evaluation was obtained in mouse models of pigmented melanoma, in which higher ^18^F-MEL050 uptake was observed in sub-millimeter pulmonary metastasis compared to ^18^F-FDG [97].

## 11. Nicotinamide Targeting

Nicotinamide is a melanin-specific agent, acting via the metabolic pathway related to tryptophan metabolism. Nicotinamide deamidation produces nicotinic acid which binds endogenous ligands HCAR2 and HCAR3 [115]. Data pertaining to nicotinamide imaging are primarily preclinical, and its role in prognostication has yet to be evaluated in the clinical setting and it is not FDA approved for PET-CT imaging in humans. Notable advantages of nicotinamides include early tumor uptake at 2 hours, with additional benefits of renal excretion and a short radiosynthesis time.

As detailed above, overexpression of melanin due to elevated tyrosine kinase activity means that it is an attractive target for both imaging and therapy [116,117]. As discussed above, labeled benzamide and its derivatives are established probes that bind to melanin both in vivo and in vitro and show high tumor accumulation and retention. However, a limitation of these benzamide-based probes is their clearance via the hepatobiliary system resulting in significant retention in some normal organs, particularly in both the liver and gastrointestinal tract, which could potentially limit detection of abdominal lesions including small bowel and liver metastases, which are not uncommon in melanoma patients [104,118,119].

Recently, attempts to overcome this limitation and improve tumor-to-background ratios of melanoma lesions on PET have resulted in the synthesis of radiolabeled nicotinamide analogues, including ^18^F-6-fluoro-N-((2-(diethylamino)ethyl)pyridine-3-carboxamide (^18^F-MEL050), described above [98,120], as well as ^123^I-N-(2-(diethylamino)ethyl)-5-iodonicotinamide (^123^I-MEL008) [121], ^131^I-Iochlonicotinamide (^131^I -ICNA) [114], ^18^F-N-(2-(diethylamino)- ethyl)-6-fluoronicotinamide (^18^F-2), and ^131^I-iodofluoronicotiamide benzamide (^131^I-IFNABZA) [122]. These agents show early and high tumor uptake and renal excretion, which is more rapid than benzamides, in part due to the enhanced hydrophilicity of their pyridine nitrogen. An additional benefit of nicotinamides includes a shorter ‘one-step’ radiosynthesis time. Whereas benzamides require multistep synthesis to incorporate a variety of radiohalogens directly onto the nicotinamide molecule ring, activation of the nicotinamide pyridine ring permits nucleophilic substitution reactions allowing for a more rapid radiosynthesis. For example, the radiosynthesis time for N-[2-(diethylamino)-ethyl]-4-^18^F-fluorobenzamide (^18^F-FBZA) is 3 h. Clearance via urinary excretion is more rapid for N-alkyl-nicotinamides ^123^I-1, ^123^I-2 and ^123^I-3 than for piperazinyl derivatives ^123^I-4 and ^123^I-5. For example, the urinary excretion of ^123^I-iodo-N-alkyl-nicotinamides is 66% injected dose (ID) is within 1 hour compared to ^123^I-benzamides (60–80% ID in 24 hours) [98,120,123]. In addition, radiolabeled nicotinamides demonstrate high radiochemical stability and favorable pharmacokinetic properties, making them attractive agents for clinical translation [114].

Synthesis of ^18^F- nicotinamides can be achieved via direct nucleophilic substitution of a halogen derivative using a radiofluorination reagent such as Kryptofix (Hexaoxa-l-10-diazabicyclo[8.8.8]hexacosane) [124] or by using the AllInOne™ module [97]. Fluorination typically involves treating a precursor molecule with a complex of M+^18^F to generate a “naked fluoride” which can then undergo nucleophilic substitution. Radiosynthesis time is typically about 40–60 min with a radiochemical yield greater than 50%. A typical tumor uptake at 2 hours is 9% ID/gram, with a tumor:blood ratio of about 60. Optimization of nicotinamides radiolabeled with halogens (including ^18^F, ^76^Br, ^123^I, ^124^I, and ^131^I) for the purpose of both PET and SPECT imaging in melanoma patients can be achieved by incorporating alkyl amide side chains allowing for their optimal binding to melanin [125].

Further research involved the conjugation of nicotinamide with ^131^I-MIP-1145 to form ^131^I-iodofluoronicotiamide benzamide (^131^I-IFNABZA) and evaluated its biological characteristics in melanoma-bearing mouse models. In cellular uptake studies, the accumulation of ^131^I-IFNABZA in melanotic B16F10 cells was almost five times higher than that in amelanotic A375 cells after a two-hour incubation time, which is consistent with rapid and strong binding to melanin. In addition, the authors calculated the absorbed dose in normal organs using the OLINDA software and found that ^131^I-IFNABZA exhibited high tumor-to-muscle ratio. The authors concluded that ^131^I-IFNABZA demonstrated a potential use for ^131^I-IFNABZA as a theranostic agent in patients with metastatic melanoma [124]. Despite these advances, researchers have yet to progress nicotinamide targeting agents to clinical translation for the molecular targeted melanoma PET-CT imaging.

## 12. MEK Targeting

MEK PET imaging can be used specifically for melanoma given that MEK is directly downstream of BRAF, which is constitutively activated in melanoma in more than 50% of cases. It can be used for imaging both primary and metastatic disease and to assess tumor heterogeneity and dose-targeted radiotherapy accordingly. Mitogen-activated protein kinase (MAPK) kinase (MEK) is an enzyme that phosphorylates MAPK as part of the cell proliferation pathway and is directly downstream of BRAF [126]. It was first identified as a key pathway in melanoma through the demonstration that the temporal activation of MEK results in transient activation of ERK, initiating signal transduction and subsequently resulting in activation of downstream modulators of cell proliferation, differentiation, survival, adhesion, angiogenesis, and cell motility [127,128]. This led scientists to target this pathway as an anti-cancer strategy in melanoma. Trametinib was the first FDA approved MEK inhibitor. This novel anticancer therapy serves as an inhibitor of MEK1 and MEK2, leading to reduced cell proliferation [129]. Though efficacious, trametinib can have severe side effects, including diarrhea, heart failure, alterations of blood glucose levels, and fever [130], making accurate dosing a key strategy to balance the risk–benefit ratio. Trametinib is also known to cause skin toxicities, most commonly on the face, chest, and back due to the sebaceous glands found preferentially in these areas [131].

Researchers sought to develop radiolabeled trametinib in order to risk-stratify patients into those who would benefit most from treatment with this therapy, similar to what has been done in other cancers and with other theranostics. ^124^I-trametinib PET imaging was evaluated to monitor MEK1/MEK2 levels in a panel of cancer cell lines and in a murine B16F10 model of melanoma [124]. The study demonstrated that ^124^I-trametinib was taken up at higher rates in BRAF and KRAS mutant cancers compared to wild type and that peak tumor concentrations in the tumor occurred at approximately 24–48 hours, compared to peak uptake in the skin and gastrointestinal tract which occurred between 6 and 24 hours. This led researchers to conclude that ^124^I-trametinib PET imaging had potential for clinical translation to personalize trametinib dosing in melanoma [132]. While initial results were promising, more studies of ^124^I-trametinib PET imaging are needed before approval for widespread clinical use.

## 13. Integrin αvβ3 Targeting

Integrin is a transmembrane receptor expressed on angiographic endothelial cells, which are players in the neovascularization process. αvβ3 integrin PET and SPECT imaging can be used for multiple tumor types given that it visualizes neovasculation beds surrounding and infiltrating tumors. Beyond tumor uptake, the highest uptake is evident in the urogenital tract, liver, spleen, and gut. ^18^F-galacto-RGD is a safe and effective tool for αvβ3 imaging in humans.

Integrin αvβ3 is a transmembrane receptor comprised of α and β subunits which is abundantly expressed on angiogenic mammalian endothelial cells [133]. αvβ3 integrin plays a significant role in neovascularization surrounding a growing tumor and initiates cell signaling cascades leading to cell migration and proliferation [134]. Specific to melanoma, αvβ3 integrin signaling promotes the transition from radial to vertical growth, resulting in penetration of the basement membrane, a critical threshold in progression to metastatic disease [135]. Due to the abundant expression of αvβ3 integrin in tumors, αvβ3 integrin-based PET imaging agents have been developed for melanoma imaging.

Tumor-induced angiogenesis can be disrupted by peptides containing the arginine-glycine-aspartic (RGD) amino acid sequence. Researchers initially discovered that the extracellular matrix protein fibronectin mediates cell attachment and demonstrated that the RGD on certain matrix proteins, mainly the fibronectin sequence, acts as a ligand for αvβ3 integrin [136]. The cell attachment activity of fibronectin can be mimicked with cyclical synthetic peptides containing the RGD sequence. Research has demonstrated that bioengineered peptides take advantage of the selective expression of αvβ3 for imaging and the delivery of therapeutic agents at neovascularization sites. The selective expression of αvβ3 shows accumulated RGD-targeted nanoparticles in angiogenic vascular beds and minimal accumulation in peripheral beds [137]. This selective expression allows for targeting of tumors while sparing healthy tissue.

Multiple radiolabeled cyclic RGD peptides have been developed and tested for imaging integrin αvβ3-positive tumors using both PET and SPECT in both preclinical and clinical investigations. One of the most widely used αvβ3 PET tracers is ^18^F-Galacto-RGD. ^18^F-Galacto-RGD is composed of the arginine-glycine-aspartic amino acid sequence conjugated with galactose and radiolabeled with ^18^F [138,139,140]. Given that αvβ3 fundamentally reflects pro-angiogenic signaling, αvβ3-PET imaging reflects the degree of angiogenesis associated with the tumor. In light of the great potential of αvβ3-PET imaging, groups sought to modify the RGD compound to optimize imaging characteristics. Research demonstrated excellent *in vivo* pharmacokinetics using ^18^F labeling of GD-containing glycopeptide cyclo(Arg-Gly-Asp-D-Phe-Lys) with 4-nitrophenyl 2 ^18^F-fluoropropionate, leading authors to conclude that the favorable excretion kinetics and low radiation dose made the compound well-suited for translation for clinical use [141]. In addition to oncological imaging, groups have explored potential non-oncological indications, including the successful use of ^18^F-Galacto-RGD for vascular inflammation imaging in mice with atherosclerosis [142]. Additional modifications have also been developed to better optimize αvβ3-PET imaging using an RGD analogue. A group synthesized and developed ^18^F-FPyPEGCBT-c(RGDfK) [143,144] using a streamlined radiochemical synthesis approach. Preclinical studies in murine models of glioma and ovarian cancer using ^18^F-FPyPEGCBT-c(RGDfK) PET-CT demonstrated uptake within tumors, suggesting its potential as a PET tracer in melanoma [144]. Further optimization may improve its imaging properties, thereby resulting in widespread clinical use.

^18^F-Galacto-RGD was the first RGD PET tracer tested on humans. A translational study by the group who developed ^18^F-Galacto-RGD evaluated the tumor imaging potential of the radiotracer and found that it allowed for non-invasive αvβ3 imaging in cancer patients [145]. Subsequent studies went on to demonstrate its role in cancer imaging, including in metastatic melanoma. Nineteen patients were administered an injection of 133–200 MBq ^18^F-Galacto-RGD followed by three consecutive PET-CT images. Time-activity curves and standardized uptake values (SUVs) were determined and compartmental modeling was used to evaluate the tumor-to-background activity. These data demonstrated a favorable biodistribution in humans and specific receptor binding, as corroborated by immunohistochemistry [146]. Together, these preclinical and clinical studies demonstrate that αvβ3 imaging has significant potential for tumor imaging, which could give rich information on angiogenesis, and that ^18^F-Galacto-RGD is an excellent αvβ3-PET imaging agent. αvβ3-PET imaging agent development is ongoing [147,148].

## 14. Conclusions

Melanoma is a deadly cancer with high metastatic potential. Accurate staging at initial diagnosis and monitoring for new or progressive disease are essential to guide management decisions. Metastatic disease below the resolution of traditional diagnostic imaging modalities presents a significant clinical challenge. Advances in molecular targeting and radiochemistry have allowed for the development of new targeted PET imaging agents for melanoma that may provide information on disease progression, response to therapy, tumor heterogeneity, and extent of disease below the resolution of CT imaging, thus changing the landscape of how the disease is imaged and treated. Here, we detail the preclinical and clinical development of some of the most promising agents. While FDG-PET has been used traditionally, FAP-PET imaging is an exciting new agent being used for many types of tumors including melanoma with the advantage of excellent visualization of tumors that have not traditionally exhibited high FDG activity. However, this agent is not specific to melanoma and PET uptake is therefore inherently non-specific. Melanin-targeting agents have the advantage of being specific to melanoma and could therefore be used in cases where there is a lack of diagnostic clarity regarding the origin of a new lesion. An inherent limitation of these agents is that they are dependent on the melanin content of lesions, which may be variable over time and heterogenous among lesions within the same patient. Other melanoma-specific agents have similar limitations, and differences in the ease of synthesis and availability may determine which emerge as the most clinically relevant. Further studies may establish one or more of these agents as the standard of care for melanoma imaging. Regardless of the agent, a future with better imaging and thus more accurate staging and appropriate treatment of melanoma offers the promise of better disease control and improved outcomes for patients.
